# Depression and loneliness in Jamaicans with sickle cell disease

**DOI:** 10.1186/1471-244X-10-40

**Published:** 2010-06-07

**Authors:** Monika R Asnani, Raphael Fraser, Norma A Lewis, Marvin E Reid

**Affiliations:** 1 Sickle Cell Unit, Tropical Medicine Research Institute, University of the West Indies, Mona Campus, Kingston 6, Jamaica (W.I

## Abstract

**Background:**

Sickle cell disease (SCD) is the commonest genetic disorder in Jamaica, and has life-long implications for those afflicted with it. It is well known that depression and loneliness may exist in those with chronic diseases, but the coexistence of depression and loneliness in people with sickle cell disease is not clear. The aim of this study is to determine the prevalence of and factors associated with depression and loneliness in the Jamaica Sickle Cell Cohort Study and its age and sex matched controls.

**Methods:**

277 patients with SCD and 65 controls were administered a questionnaire that studied demographics, disease severity, depression, and loneliness. Regression analyses were done to examine relationships between outcomes and associated variables.

**Results:**

Depression was found in 21.6% of patients and 9.4% in controls. Loneliness scores were also significantly higher in patients (16.9 ± 5.1) than in controls (14.95 ± 4.69). Depression was significantly associated with unemployment [OR = 2.9, p-value: < 0.001], whereas unemployment (p-value: 0.002), and lower educational attainment were significantly associated with loneliness.

In patients with SCD, depression was significantly associated with being unemployed (OR 2.4, 95% CI 1.2,4.6, p-value:0.01), presence of a leg ulcer (OR = 3.8, 95% CI: 1.7, 8.4, p-value: 0.001), frequent visits (OR = 3.3, 95% CI: 1.2, 8.9, p-value: 0.019), and frequent painful crises (OR = 2.5, 95% CI: 1.1, 5.8, p-value: 0.035). Not being employed (Coef.: 2.0; p-value: 0.004) and higher educational attainment (tertiary vs. primary education, Coef.: -5.5; p-value: < 0.001) were significant associations with loneliness after adjusting for genotype.

**Conclusions:**

Health workers need to actively look for and manage these problems to optimize their patients' total biopsychosocial care.

## Background

Sickle Cell Disease (SCD) is a chronic and potentially, quite a debilitating disease. The disease is severe and may result in significant morbidity, as well as a shortened life span. There is considerable variability in the clinical course of sickle cell disease with some patients experiencing few to no complications and others having multiple organ involvement [1]. Painful crises, lung diseases such as Acute Chest Syndrome, leg ulcerations, and priapism are common complications of the disease in Jamaica. These and other complications contribute a lot to the social isolation and lack of normal functioning in these patients.

Depression is one of the most common complications of chronic illnesses [2-4]. According to one survey [2], depression was found to be common in patients diagnosed with: recent heart attacks (45%), hospitalized cancer patients (42%), recent stroke survivors (40%), and people with multiple sclerosis (40%), Parkinson's disease (40%), and diabetes (33%). Depression caused by chronic illness often aggravates the illness, especially if the condition causes pain, fatigue, or disruption of social life.

The prevalence of depression in SCD patients has been reported to be high [5-9]. The Pain in Sickle Cell Epidemiology Study has also shown the prevalence of depression (as assessed by studying daily diaries) to be a high of 27.6% [10]. Hilton *et al *(1997) have reported on the prevalence of overall psychiatric morbidity in patients with SCD in Jamaica but not of depression [11].

Loneliness is a measure of being cut off or separated from others. It refers to a deficiency of social contact compared to what is desired [12], and is fundamentally an aversive and distressing experience with potentially serious consequences [13]. As such it is a distinct psychological problem from depression. Loneliness has been linked to a number of somatic and psychological difficulties and has been associated with lower reported life satisfaction, suicide and alcoholism [14]. A few studies have shown loneliness to be increased in patients with acute or chronic diseases [15-17]. Loneliness and depression also have been found to co-exist in a number of populations [18-20]. In SCD, depression and loneliness may be as a result of living with a chronic disease itself, or may be a result of societal and health care providers' response to these persons [9,21].

No studies have looked at the co-existence of depression and loneliness in SCD and it is hypothesized that depression and loneliness will have a high prevalence and coexist in this population. In this study therefore, we have sought to determine the prevalence of depression and loneliness in a birth cohort of patients with SCD in Jamaica and compare it to its age- and sex-matched set of controls. We have also sought to determine possible socioeconomic and clinical factors associated with depression and loneliness in this disease group.

## Methods

### Sampling

The Jamaica Sickle Cell Cohort Study (JSCCS) incorporates all patients with SCD detected during the screening of 100,000 consecutive deliveries at the Victoria Jubilee Hospital - the country's main government maternity hospital - from June 1973 to December 1981. This screening identified 552 cases with SCD, 315 children with homozygous sickle cell (SS) disease, 178 with sickle cell-haemoglobin C (SC) disease, 33 with sickle cell-beta+ thalassaemia (S beta+ thal) and 14 with sickle cell-beta^0 ^thalassaemias (S beta^0 ^thal). These patients have been closely followed since birth and are seen at the only comprehensive sickle cell clinic (SCU) located at the University of the West Indies in Kingston, Jamaica for routine health maintenance checks and for all significant sick events in an effort to document the natural history of the disease. They are actively located if they have not presented to the SCU for more than 6 months- 1 year. 277 patients were available (the rest having been lost mainly to emigration and death), and all took part in the study where they were administered the study questionnaire comprising questions on socio-demographics, disease severity, depression and loneliness.

At the initial recruitment of the JSCCS, the first 125 SS babies were each matched with two controls with an AA phenotype of the same sex born closest in time (usually immediately before and after) resulting in 250 controls collected over the first 21/2 years. 64 of these controls are still available and were also administered the questionnaire including questions on socio- demographics, depression and loneliness.

### Data collection

The demographic data collected included age, sex, marital status, genotype, employment status, and the highest educational level they had attained. Questions were also included on their utilization of the clinic (SCU) services, frequency of painful crises, and whether they had leg ulceration as a result of their sickle cell disease. Pain was defined as 'severe bony pains requiring opiod analgesia for relief', and was categorized as being 'Rare' if they rarely had bony pains or 'Frequent' if they suffered a painful crisis at least once monthly. Similarly, utilization of the clinic services was categorized as '≤ 1-3 times/year' (Rare) or '≥ once/month' (Frequent). Depression was measured using the 21-point Beck Depression Inventory-version II (BDI), and loneliness using the UCLA-8 Loneliness scale (UCLA).

#### Beck Depression Inventory-II

The BDI is one of the most commonly utilized measure of depression among adolescents and adults by clinicians and researchers alike [22]. The BDI-II has demonstrated high internal consistency, good test-retest reliability, and good construct and concurrent validity with other common measures of depression in clinical and nonclinical samples [23]. Discriminant validity has also been demonstrated through weaker relationships with measures of other psychopathology, such as anxiety [24]. In Jamaica, the BDI has been validated previously in a sample of university students [25].

#### UCLA-8 Loneliness Scale

A widely used loneliness measure is the UCLA loneliness scale developed by Russell & colleagues [26,27]. Psychometric analyses including confirmatory factor analyses have confirmed that this is a reliable and valid measure of loneliness across a variety of populations [28-30]. Hays and DiMatteo [12] have developed an eight-item short form of the UCLA-20, the UCLA-8, which has been shown to be highly correlated with the original form (*r *= 0.91). Scores on the UCLA-8 are also well distributed, ranging from 0% to 100% of the maximum possible. The UCLA-8 is a practical alternative to the UCLA-20 and reduces respondent burden. The latter may lead to improved quality of data being collected. Although the estimated reliability of the UCLA-8 is a little lower than that of the UCLA-20, there is greater homogeneity in this measure [12]. Cross-cultural studies have shown the stability of the measure across countries and cultures [31,32].

The controls were given the questionnaire that included information only on socio-demographics as well as the BDI and UCLA. Both instruments showed acceptable internal consistency reliabilities in SCD (BDI: α = 0.95; UCLA: α = 0.82) as well as in the AA (BDI: α = 0.90; UCLA: α = 0.78) group. Higher scores on the BDI and UCLA imply worsening depression and loneliness respectively.

Written, informed consent was obtained prior to administering the questionnaire that had been pretested. Complete confidentiality was maintained throughout the study. The study was granted ethical approval by the University of the West Indies/University Hospital of the West Indies Ethical Committee.

### Statistical analyses

Descriptive statistics are reported as frequency and percent for categorical data and as mean and SD for continuous data. Continuous variables were compared by Student's *t *test. Pearson correlations were used to assess linear associations between the two scales. Age, gender, highest educational level achieved, group (SCD vs. AA), employment status, frequency of visit to SCU, pain crises frequency and other symptoms characteristic of SCD were used as covariates in our regression models. Depression and loneliness were the outcomes of interest. The analyses were first done on the entire group then on the SCD subgroup. As the author Aaron Beck has stated that the BDI has no stringent cut-offs, a higher score of 17 was used to allow greater specificity to diagnosis. Also, in chronic illnesses that have somatic symptoms, the BDI may give falsely elevated scores [33,34], and as SCD does cause symptoms such as fatigue and loss of energy, a higher cut-off of 17 was utilized. Logistic regression was used to examine the relationship between independent variables and depression, and multivariate linear regression to examine the possible factors associated with loneliness. Data was analyzed using Stata Software version 10 for Windows™ (StataCorp, College Station, TX).

## Results

The study sample consisted of 277 patients with SCD (mean age 31 years) and 65 patients with normal AA genotype (mean age 33.6 years), with equal proportions of males and females in each subgroup (Table 1). A significantly greater proportion of the controls had higher levels of educational attainment and was employed.

**Table 1:** Summary of patient characteristics

	SCD (N = 277)	AA (N = 65)
Age in years, mean (SD)***	31.0 (2.50)	33.6 (0.67)
Gender (female/male)	140/137	37/28
Employment (yes/no)***	157/107	58/7
Educational status**		
Primary	35	-
Secondary/High	121	34
Post Secondary/Skills training	84	18
Tertiary	24	12
Depression score*, mean (SD)	9.19 (10.38)	6.42 (7.94)
Proportion depressed*	21.6%	9.4%
Loneliness score**, mean (SD)	16.94 (5.10)	14.95 (4.69)

p < 0.05, ** p < 0.01, *** p < 0.001

The mean depression score (9.19 ± 10.4, Range 0, 50) was significantly higher in SCD than in AA controls (6.4 ± 7.9, Range 0, 41), with almost twice the proportions of SCD persons being depressed as compared to the controls. The mean loneliness score was also significantly higher in SCD (16.9 ± 5.1) than in AA controls (14.95 ± 4.69). The Pearson's correlation between the two scales was 0.39 for those with SCD implying that the two scales were probably not measuring a similar concept.

**Figure 1:**
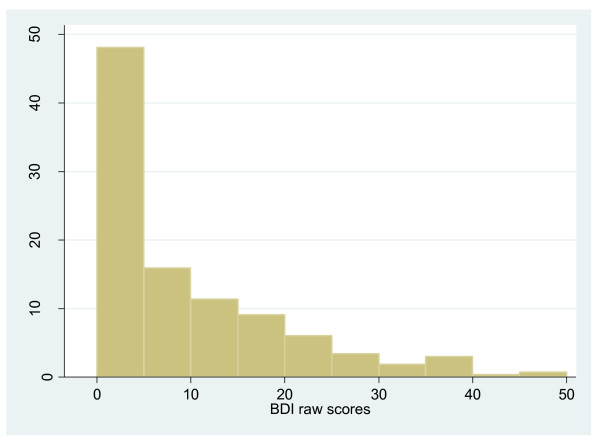
**Distribution of BDI scores in SCD**.

The distribution of BDI scores in those with SCD is shown in Figure 1. Further comparisons were done within the SCD cohort to determine differences between those who were not depressed (BDI scores (mean ± SD): 4.6 ± 4.7) and those depressed (BDI scores (mean ± SD): 25.8 ± 8.2) (Table 2). There were no differences in age, sex, genotype and marital status of those 'not depressed' and those 'depressed'. However, depressed patients had significantly lower levels of educational attainment (p-value 0.002) and more were unemployed (p-value 0.001). Those depressed had worse disease as evidenced by having higher proportion of leg ulceration (p-value 0.006), higher frequency of painful crises (p-value < 0.001) and more frequent visits to the SCU (p-value < 0.001). The depressed patients also had significantly higher mean loneliness scores (mean score: 16.1 vs. 20.1, p-value < 0.0001).

**Table 2:** Sociodemographic and clinical variables in persons with SCD

	Nil Depression	Depression	p-value
Age in years, mean(SD)	30.9 (2.5)	31.1(2.5)	0.582
			
Sex, Male:Female	105:102	23:34	0.165
			
Genotype, n (%)			0.917
SS	117 (56.5)	33 (57.9)	
SC	64 (31)	18 (31.6)	
Others	26 (12.5)	6 (10.5)	
			
Marital Status, n (%)			0.742
Single	184 (88.9)	52 (91.2)	
Married	21 (11.1)	5 (8.8)	
			
Educational Status, n (%)			0.002
Primary	26 (12.6)	9 (15.8)	
Secondary/High	85 (41.0)	36 (63.2)	
Vocation/Skills	72 (34.8)	12 (21.0)	
Tertiary	24 (11.6)	0 (0)	
			
Employment status, n (%)			0.001
Yes	134 (64.7)	23 (40.4)	
No	73 (35.3)	34 (59.6)	
			
Leg Ulcers, n (%)			0.006
No	175 (84.5)	39 (68.4)	
Yes	32 (15.5)	18 (31.6)	
			
Pain Category, n (%)			<0.001
Rare	180 (87)	36 (63.2)	
Frequent	27 (13)	21 (36.8)	
			
Visits to SCU, n (%)			<0.001
Rare (≤1-3 times/year)	192 (92.8)	42 (73.7)	
Frequent (≥Once/month)	15 (7.2)	15 (26.3)	
			
Loneliness Score, mean (SD)	16.1 (4.8)	20.1 (5.2)	<0.0001

### Depression and loneliness

The relationship between the various factors (age, gender, highest educational level achieved, group [SCD vs. AA], employment status) and depression was explored with a series of logistic regression models, and their relationship with loneliness was explored with multivariate linear regression. In the final model, not being employed was the only factor significantly associated with depression in the study sample [OR = 2.9 (95% CI: 1.6, 5.1) p-value: < 0.001], adjusting for presence of SCD (Table 3). Not being employed was also a significantly associated with loneliness (p-value: 0.002), whereas higher levels of educational attainment were associated with lower levels of loneliness (Table 4). Higher depression scores were associated with higher loneliness scores (regression coef.; 0.78; p value < 0.001).

**Table 3:** Predictors of depression adjusting for presence of disease (SCD + AA)

Variable	OR	95% CI	p-value
Not employed	2.9	1.6, 5.1	<0.001
Presence of SCD	1.9	0.8, 4.8	0.17

**Table 4:** Predictors of loneliness adjusting for presence of disease (SCD+AA)

Variable	Coefficient	95% CI	p-value
Not employed	1.96	0.7, 3.2	0.002
*Education level			
Vocation/Skills	-2.2	-4.2, -0.24	0.03
Tertiary	-6.4	-9.0, -3.8	<0.001
Presence of SCD	0.66	-0.7, 2.1	0.35

*Compared to 'Primary education'

### Depression and loneliness in SCD

The odds ratios (OR) for the factors that were significantly related to depression (Table 5), adjusting for genotype, were being unemployed (OR 2.4, 95% CI 1.2,4.6, p-value:0.01), presence of leg ulcer (OR = 3.8 (95% CI: 1.7, 8.4, p-value: 0.001), frequent visits to the sickle cell unit (OR = 3.3, 95% CI: 1.2, 8.9, p-value: 0.019), and frequent painful crises (OR = 2.5, 95% CI: 1.1, 5.8, p-value: 0.035).

**Table 5:** Predictors of depression in SCD adjusting for genotype

Variable	OR	95% CI	p-value
Not employed	2.4	1.2, 4.6	0.010
Presence of leg ulcers	3.8	1.7, 8.4	0.001
*Frequent visits to the unit (≥ once/month)	3.3	1.2, 8.9	0.019
**Frequent pains (≥ once monthly)	2.5	1.1, 5.8	0.035
***SC genotype	2.5	1.1, 5.4	0.027

• *Compared to 'Visits 1-3 times a year';

• ** Compared to 'Rare pains';

• ***Compared to 'SS genotype'

Multivariate linear regression yielded, once again, not being employed (Coef: 2.0, p-value: 0.004), and higher educational attainment (vocational/skills training compared to primary education Coef: -2.3, p-value: 0.028; tertiary education compared to primary education, Coef: -5.5, p-value: < 0.001) as factors significantly associated with loneliness after adjusting for genotype (see Table 6).

**Table 6:** Predictors of loneliness in SCD adjusting for genotype

Variable	Coefficient	95% CI	p-value
Not employed	2.0	0.6, 3.4	0.004
*Education level			
Vocation/Skills	-2.3	-4.4, -0.3	0.028
Tertiary	-5.5	-8.4, -2.5	<0.001

*Compared to 'Primary education'

## Discussion

The primary aim of this study was to examine the prevalence of depression and loneliness in adult patients with Sickle Cell Disease in Jamaica and compare it to an age-matched set of controls.

Depression was found in a significantly higher proportion of patients with SCD (21.6%) than in controls (9.4%). The latter compares well to other studies that have shown the prevalence of depression in the general Jamaican population to be at 8% [35]. Previous studies [5] using the BDI have reported prevalence of depression in SCD to be at 44%, but using a cut off score of 14. Wilson Schaeffer *et al *(1999) found the prevalence to be at 43.4%, which fell to 18% using a more stringent cut-off. They used the Centre for Epidemiologic Studies - Depression scale (CES-D) as their tool and studied 440 adults. The mean age of this sample however, was much higher at 33.7 (SD = 11.5). Most of previous studies possibly have a symptomatic bias attributable to the recruitment of patients from a clinic population. This is in contrast to our study where we have examined a well birth cohort of patients which has been followed up actively over the years. This has allowed for this study to include those people with SCD who tend to have good physical health.

Similarly, patients with SCD have shown higher levels of loneliness than their controls. To our knowledge, there are no previous reports of loneliness among this patient group. Loneliness seems to be strongly related to a broad range of negative emotional and cognitive states such as depression and anxiety [26], and it has been suggested that loneliness and depression may act synergistically to diminish well being [36]. Our study has also shown a moderately high correlation between loneliness and depression, and higher levels of loneliness among those who are depressed. Even though most studies on loneliness have been done in nursing home residents and the elderly, socially isolated young adults have shown higher rates of mortality [37,38], even after adjusting for other risk factors for death. Lonelier persons have shown lower cardiovascular function (higher total peripheral resistance and lower cardiac output) [39], as well as lower immune and higher stress levels [40]. It remains to be seen if loneliness may lower immune response, cardiovascular resilience, etc. in patients with SCD.

It is important to note that lack of employment was a significant factor associated with higher levels of depression and loneliness irrespective of having SCD or not. Also, those with lower levels of educational attainment tended to be lonelier. Previous studies have also confirmed that poorer social and economic circumstances lead to higher levels of depression, as well as worse disease outcomes [8,10,41]. It is important against this background that health care professionals remain vigilant to proper school attendance and educational attainment of young children with SCD, so as to enable them for better employment opportunities in the future.

### Depression, loneliness and SCD

Another aim of the study was to examine variables that were significantly associated with depression and loneliness in those with SCD. Being unemployed, having leg ulcerations, more frequent painful crises, and frequently visiting SCU for health care were all positively associated with depression in those with SCD. Once again, unemployment and lower educational attainment were associated with greater loneliness scores among this disease population. Gender, age and marital status have shown no associations with either of these outcomes.

More frequent painful crises and visiting the unit more frequently were positively associated with depression. Depressive symptoms often complicate chronic pain, and may lower the threshold and tolerance for pain. These observations have been consistent in previous studies [5,8,10,42]. Our study has also shown leg ulceration to be significantly associated with depression and it was a frequent complication of SCD in this study population (18.9%). Although leg ulceration is not a complication that is associated with high mortality in SCD, it is a frequent and chronic condition that leads to a huge degree of morbidity especially in younger people with SCD [43]. These persons have also been shown to have lower educational attainment and inability to form stable marital relations [44].

A surprising finding from this study was the fact that heterozygous SC disease (as compared to SS Disease) was significantly associated with greater depression (OR 2.5, 95% CI 1.1,5.4, p-value:0.027), even though both SS and SC have demonstrated same frequency of depression (22%) in this study sample. This is despite the fact that those with SC disease were more likely to be employed, and had less severe disease as evidenced by less frequent visits to the unit, less frequent painful crises and lower prevalence of leg ulceration (results not shown). This finding may point to lower coping mechanisms in those with the less severe form of disease; past findings have also similarly pointed to greater psychological distress among those with milder genotypes of SCD [8,45]. Severity of disease is not always correlated to psychological adjustment or coping, and maybe those with more severe illness have better social support systems and may have learnt better stress-management strategies to help them with their illness [45]. Further studies would enable us to gain understanding into mechanisms of depression in this disease population and how these may vary among different genotypes.

The main limitations of this study relate to the instrument used to assess depression and the characteristics of the sample studied. Even though the BDI is a valid instrument [46] for measuring the degree of depression, a structured clinical interview would have been more diagnostic. The second limitation is that the study was conducted in the cohort where the oldest person studied was 35 years of age. The study needs to be extended to older age groups as well. A bias that using the cohort has introduced in the study is the survival bias, as those who had been lost due to mortality and migration were missed out in the study, and these individuals may have had more severe (in case of those who died) or variable severity (in case of those who migrated) disease.

## Conclusions

This study confirms the fact that SCD is a chronic disease where the prevalence of depression and loneliness increases to higher levels than in the general population. The depressive symptoms in this population show significant association with demographic variables such as educational status, and employment. Health care professionals working with the SCD population need to be aware of these factors. They need to ensure continuing education of the patients, and hence increase further their ability to have worthwhile employment. These factors need to be at the forefront of all health surveillance visits from childhood. Even though a myriad of studies have shown that chronic diseases often co-exist with depression, sadly this evidence has not translated into practice [47], as these feelings of depression, anxiety etc. may be trivialized or ignored in the bigger picture of managing the 'main' illness. Co-existing depression needs to be diagnosed and managed optimally for total biopsychosocial care of the patient, especially as there appears to be a bi-directional relationship between depression and many medical illnesses.

Chronic complications such as frequent painful crises and leg ulcers, which in turn increase healthcare utilization by those with these complications, are also issues that need to be recognized. More effective pain relief would no doubt benefit those with depression. Conversely, having concurrent depression may worsen pain, and so use of antidepressants may be considered beneficial as adjunct therapy in pain management.

## Competing interests

The authors declare that they have no competing interests.

## Authors' contributions

MA contributed to study design, data collection, analysis and manuscript preparation; RF contributed to study design and data analysis; NL contributed to data collection and final manuscript preparation; MR contributed to design, data collection, analysis and manuscript preparation. All authors have read and approved the final manuscript.

## Pre-publication history

The pre-publication history for this paper can be accessed here:

http://www.biomedcentral.com/1471-244X/10/40/prepub
